# Supplementary material for: Insights into the RNA Virome of the Corn Leafhopper Dalbulus maidis, a Major Emergent Threat of Maize in Latin America
Source: Viruses. 2024 Oct 9;16(10):1583. doi: 10.3390/v16101583 (PMC11512364; doi:10.3390/v16101583)
Supplement: Supplementary file 1 [file viruses-16-01583-s001.zip › Supp Table 1_HD.pdf]

Supplementary Table S1. NCBI publicly available datasets used for virus discovery. Total virus reads and per million (RPM) are based on mapping results using Bowtie.2 with standard parameters against the genome consensus sequences of Chicharrita del maiz orthomyxo-like virus 1 (ChMOMLV), Chicharrita del maiz beny-like virus 1 (ChMBLV), Chicharrita del maiz rhabdo-like virus 1 (ChMRV), Chicharrita del maiz iflavirus 1 (ChMIfV1), Chicharrita del maiz iflavirus 2 (ChMIfV2), and Chicharrita del maiz bunyan-like virus 1 (ChMBV).

Run	BioProject	BioSample	Experiment	Country	Total library reads	ChMOMLV reads	ChMOMLV RPM	ChMBLV reads	ChMBLV RPM	ChMRV reads	ChMRV RPM	ChMIfV1 reads	ChMIfV1 RPM	ChMIfV2 reads	ChMIfV2 RPM	ChMBV reads	ChMBV RPM
SRR8248833	PRJNA505618	SAMN10430478	SRX5066649	Argentina	44568016	**2**	0,0	0	0,0	**6720**	**150,8**	**151**	**3,4**	0	0,0	0	0,0
SRR10334087	PRJNA562189	SAMN13091747	SRX7044228	USA	210894690	0	0,0	0	0,0	0	0,0	0	0,0	**10846**	**51,4**	0	0,0
SRR15166998	PRJNA562189	SAMN20251994	SRX11474353	USA	45974618	**3020**	**65,7**	**13192**	**286,9**	0	0,0	**16114**	**350,5**	**680**	**14,8**	0	0,0
SRR1821981	PRJNA272239	SAMN03341953	SRX893575	USA	697217	**172**	**246,7**	0	0,0	0	0,0	0	0,0	0	0,0	**540**	**774,5**
SRR5010611	PRJNA318849	SAMN06009808	SRX2341563	Brazil	44939338	**949**	**21,1**	0	0,0	0	0,0	**19631**	**436,8**	**1114**	**24,8**	**363**	**8,1**
SRR5010612	PRJNA318849	SAMN06009807	SRX2341564	Brazil	45008074	**397**	**8,8**	0	0,0	0	0,0	**11422**	**253,8**	**180**	**4,0**	**388**	**8,6**
SRR5010613	PRJNA318849	SAMN06009806	SRX2341565	Brazil	43120958	0	0,0	0	0,0	0	0,0	**9596**	**222,5**	**619**	**14,4**	**573**	**13,3**
SRR11822333	PRJNA631706	SAMN14890635	SRX8372961	USA	36054352	0	0,0	0	0,0	0	0,0	0	0,0	**10732**	**297,7**	0	0,0
SRR11822334	PRJNA631706	SAMN14890635	SRX8372960	USA	42673870	0	0,0	0	0,0	0	0,0	0	0,0	**23298**	**546,0**	0	0,0
SRR11822335	PRJNA631706	SAMN14890505	SRX8372959	USA	41853828	0	0,0	0	0,0	0	0,0	0	0,0	**6677**	**159,5**	0	0,0
SRR11822336	PRJNA631706	SAMN14890505	SRX8372958	USA	51372620	0	0,0	0	0,0	0	0,0	0	0,0	**4168**	**81,1**	0	0,0
SRR11822337	PRJNA631706	SAMN14890505	SRX8372957	USA	40536434	0	0,0	0	0,0	0	0,0	0	0,0	**10398**	**256,5**	0	0,0
SRR11822338	PRJNA631706	SAMN14890505	SRX8372956	USA	24793552	0	0,0	0	0,0	0	0,0	0	0,0	**12651**	**510,3**	0	0,0
SRR11822339	PRJNA631706	SAMN14890446	SRX8372955	USA	34075044	0	0,0	0	0,0	0	0,0	0	0,0	**10623**	**311,8**	0	0,0
SRR11822340	PRJNA631706	SAMN14890446	SRX8372954	USA	45038780	0	0,0	0	0,0	0	0,0	0	0,0	**4127**	**91,6**	0	0,0
SRR11822341	PRJNA631706	SAMN14890756	SRX8372953	USA	46079864	0	0,0	0	0,0	0	0,0	0	0,0	**24639**	**534,7**	0	0,0
SRR11822342	PRJNA631706	SAMN14890756	SRX8372952	USA	36986548	0	0,0	0	0,0	0	0,0	0	0,0	**14839**	**401,2**	0	0,0
SRR11822343	PRJNA631706	SAMN14890756	SRX8372951	USA	42869668	0	0,0	0	0,0	0	0,0	0	0,0	**37763**	**880,9**	0	0,0
SRR11822344	PRJNA631706	SAMN14890756	SRX8372950	USA	38197304	0	0,0	0	0,0	0	0,0	0	0,0	**23300**	**610,0**	0	0,0
SRR11822345	PRJNA631706	SAMN14890635	SRX8372949	USA	48481712	0	0,0	0	0,0	0	0,0	0	0,0	**16570**	**341,8**	0	0,0
SRR11822346	PRJNA631706	SAMN14890635	SRX8372948	USA	42292438	0	0,0	0	0,0	0	0,0	0	0,0	**11644**	**275,3**	0	0,0
SRR11822347	PRJNA631706	SAMN14890446	SRX8372947	USA	43790788	0	0,0	0	0,0	0	0,0	0	0,0	**2662**	**60,8**	0	0,0
SRR11822348	PRJNA631706	SAMN14890446	SRX8372946	USA	22991808	0	0,0	0	0,0	0	0,0	0	0,0	**10249**	**445,8**	0	0,0
